# A case of successful anesthetic management in a patient with Trousseau’s syndrome who underwent surgery for malignant ovarian tumor

**DOI:** 10.1186/s40981-020-00339-2

**Published:** 2020-05-09

**Authors:** Yasuhiro Watanabe, Kayoko Matsunuma, Toru Kaneda

**Affiliations:** Department of Anesthesia, Japanese Red Cross Shizuoka Hospital, 8-2 Oute-machi Aoi-ku, Shizuoka, 420-0853 Japan

**Keywords:** Trousseau’s syndrome, Cancer-associated thrombosis, Cerebral infarction, Anticoagulant therapy, Anesthetic management

## Abstract

**Background:**

Trousseau’s syndrome, also known as cancer-associated thrombosis, has several perioperative considerations, including the timing of surgery, anticoagulant therapy, and anesthetic technique. While appropriate anesthetic management is critical, few clinical reports have addressed the issue. Here, we report a patient with Trousseau’s syndrome who successfully underwent gynecological surgery 1 month after a massive cerebral infarction.

**Case presentation:**

A 46-year-old woman with malignant ovarian tumor and deep venous thrombosis developed systemic thromboembolism, including a massive right cerebral infarction, despite receiving direct oral anticoagulant therapy. She was diagnosed with Trousseau’s syndrome and was transferred to our hospital 17 days after the onset of cerebral infarction with left incomplete hemiparesis. Semi-radical gynecological surgery was scheduled in another 14 days (31 days after the cerebral infarction). A temporary inferior vena cava filter was placed, and both direct oral anticoagulant and antiplatelet drugs were substituted with unfractionated heparin infusion. She underwent surgery uneventfully under general anesthesia with desflurane and remifentanil. Postoperative analgesia was achieved with a peripheral nerve block and continuous intravenous infusion of fentanyl. The tumors were fully resected, thereby only anticoagulant therapy for residual venous thrombus was continued. She had a good perioperative course and was discharged without cerebral complications or thromboembolism.

**Conclusions:**

In patients with Trousseau’s syndrome, both early radical surgery and preventing perioperative cerebrovascular complications are critical. In our present case, Trousseau’s syndrome was successfully operated under general anesthesia 1 month after a massive cerebral infarction.

## Background

Since Trousseau first reported migratory thrombophlebitis as a concomitant medical condition in patients with advanced gastric cancer [1], the link between coagulation disorders and malignancy has received more attention from clinicians and researchers [2]. Recently, Trousseau’s syndrome has been redefined as cancer-associated thrombosis and is characterized by systemic, particularly cerebral, thromboembolism caused by hypercoagulability associated with malignancy [3]. There are certain perioperative concerns for patients with Trousseau’s syndrome (i.e., cancer patients with thromboembolism), including the timing of radical surgery, administration of anticoagulant therapy, and anesthetic technique. Few clinical reports have discussed the appropriate anesthetic management technique for Trousseau’s syndrome. Hence, we report a case of successful anesthetic management in a patient with Trousseau’s syndrome who underwent gynecological surgery 1 month after a massive cerebral infarction.

## Case presentation

**Fig. 1:**
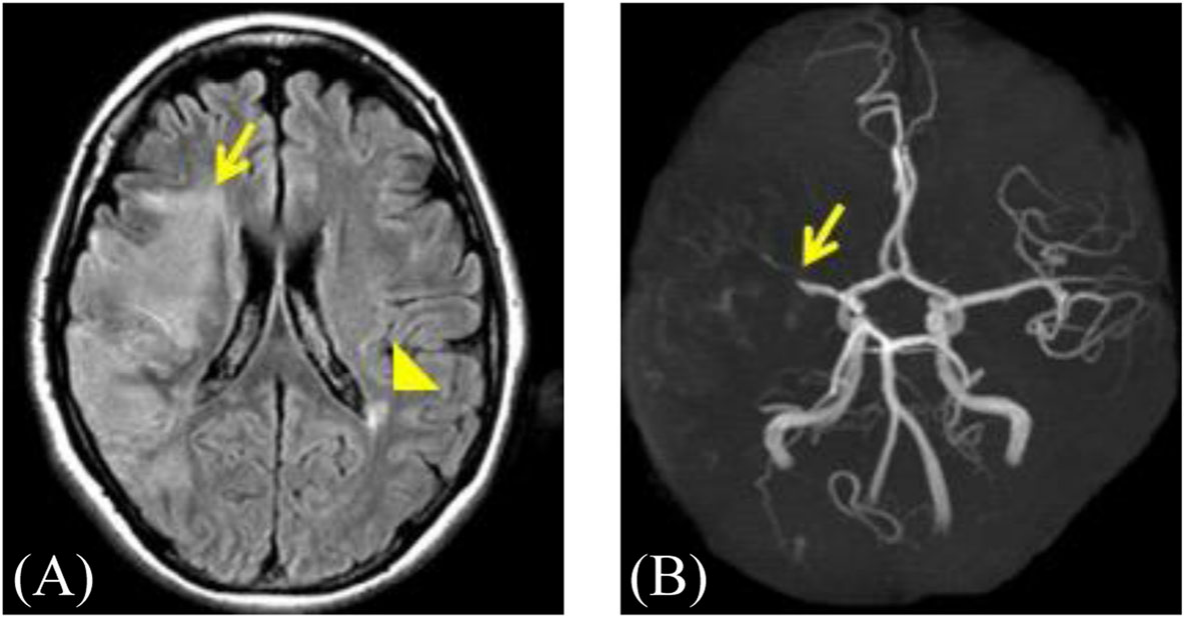
**a** Magnetic resonance imaging of the brain. Diffusion-weighted image demonstrating massive right cerebral infarction in the cerebral cortex (arrow) and another infarction (arrowhead) in the left hemisphere. **b** Magnetic resonance (MR) angiography of cerebral arteries. MR angiography demonstrating total occlusion of the M1 segment in right middle cerebral artery (arrow)

A 46-year-old woman (height; 172 cm, body weight; 54 kg) visited a neighborhood hospital complaining of abdominal distension. She had no previous history except heminephrectomy for renal angiomyolipoma. Magnetic resonance imaging (MRI) revealed a large left ovarian tumor. Together with markedly elevated tumor marker levels; carbohydrate antigen (CA) 19–9 of 606 U/mL (normal, < 37 U/mL) and CA 125 of 432 U/mL (normal, < 35 U/mL), malignant ovarian tumor was suspected and surgery was planned. Anticoagulant therapy with edoxaban 30 mg/day was started because of remarkably increased D-dimer level (17.5 μg/mL) and an old thrombus in the left soleal vein revealed by ultrasonography. She developed acute onset of left hemiparesis 10 days later and was admitted to another hospital in an emergency. Multiple cerebral infarctions as well as total occlusion of the right middle cerebral artery were revealed by MRI and MR angiography, respectively (Fig. 1a, b). Contrast-enhanced computed tomography (CT) revealed splenic and left renal infarctions, resulting in the diagnosis of Trousseau’s syndrome. Despite the administration of tissue-plasminogen activator and endovascular thrombectomy, incomplete left hemiparesis remained, and cilostazol 100 mg/day was added to edoxaban.

She was transferred to our hospital on the 17th day, and was scheduled to undergo a semi-radical surgery for the malignant ovarian tumor on the 31st day (1 month) after the cerebral infarction. To reduce the relative risk of pulmonary embolism, a temporary inferior vena cava (IVC) filter (Denali™ Vena Cava Filter, BD, Franklin Lakes, NJ, US) was placed on the 2nd day after admission. Both edoxaban and cilostazol were discontinued 5 days preceding surgery, and intravenous (IV) infusion of unfractionated heparin was initiated on the next day. Preoperative electrocardiogram showed regular sinus tachycardia. Transthoracic echocardiography (TTE) and carotid artery echography did not detect causative embolic sources or abnormalities such as an intracardiac right to left shunt. Blood test results the day before surgery were not remarkable except activated partial thromboplastin time (APTT) of 40 sec, fibrinogen of 656 mg/dL, and D-dimer of 1.4 μg/mL. Heparinization was terminated 6 h prior to surgery. In addition to standard monitoring, we observed depth of anesthesia (BIS™, Medtronic, Minneapolis, MN, USA), and regional cerebral oxygen saturation levels (rSO_2_; INVOS™ 5100C, Edwards Lifesciences, Irvine, CA, USA) during anesthesia. The baseline rSO_2_ at room air was 58% and 66% on the right and left side of the forehead, respectively. Transesophageal echocardiography (TEE) was kept at hand in preparation for hemodynamic instabilities such as pulmonary embolism.

General anesthesia was induced with propofol 60 mg and rocuronium 40 mg after starting infusion of remifentanil at 0.4 μg/kg/min and the trachea was intubated. Anesthesia was maintained with desflurane and continuous infusion of remifentanil to maintain bispectral index between 40 and 60. An arterial pressure catheter was inserted in the right radial artery, followed by an ultrasound-guided transversus abdominis plane block and rectus sheath block with 0.125% levobupivacaine. Rocuronium bromide was added to keep the train-of-four visible twitch at less than 1 count, and IV infusion of fentanyl set at 25 μg/h was initiated about 30 min after the start of surgery for postoperative patient-controlled analgesia (PCA). Intraoperative respiratory and circulatory dynamics were relatively stable, and low BP requiring intervention only occurred twice. A single bolus injection of ephedrine 4 mg or phenylephrine 0.05 mg was used to adjust the low BP. A temporary reduction in bilateral rSO_2_ levels 54%, 58% respectively was observed before the start of surgery, which returned to normal without intervention. Bilateral rSO_2_ levels were stable thereafter, and fluctuated in the range of approximately ± 10% of its initial values. Total hysterectomy with bilateral salpingo-oophorectomy and omentectomy was accomplished uneventfully. The operation duration was 159 min, and total anesthetic time was 204 min. Along with continuous infusion, an intermittent bolus injection of fentanyl (total dose; 300 μg) resulted in the estimated effect-site concentration of 1.35 ng/mL at the end of surgery. After completion of surgery, residual muscle relaxant confirmed by neuromuscular monitoring was reversed with sugammadex 200 mg. She immediately emerged from general anesthesia with no additional neurological deficits, and was transferred to the intensive care unit. Total intraoperative bleeding was 515 g, and the hemoglobin level immediately after the surgery was 9.9 g/dL. Postoperative vital signs were also stable, and no blood products were transfused. IV infusion of heparin was restarted 6 h after surgery without increased blood drainage, which was then substituted with a subcutaneous injection on postoperative day (POD) 3. Tumors, including disseminated lesions, were considered to be fully resected, heparin administration was finally discontinued on POD 7, and edoxaban (30 mg/day) for residual venous thrombus was resumed. On POD 9, the IVC filter was removed, to which a small amount of white thrombus was attached. She was discharged on POD 18 to a rehabilitation hospital without cerebral complications such as hemorrhagic infarction and thromboembolic stroke.

## Discussion

Cerebral infarction is one of the most serious comorbidities in patients with cancer. Our patient had no history of hypertension, diabetes mellitus, or dyslipidemia, all of which are major risk factors for atherothrombotic cerebral infarction. In addition, there was no evidence of arrhythmias which might cause cardiogenic cerebral infarction due to the formation of intracardiac thrombi. Either TTE or carotid artery echography did not reveal any causative embolic sources or abnormalities. Based on these findings, we speculated that her systemic thrombosis was caused by nonbacterial thrombotic endocarditis associated with Trousseau’s syndrome [4]. Trousseau’s syndrome is most commonly associated with gynecological cancers, and other malignancies including renal and gastrointestinal cancers would be the primary lesions, most of which are mucin-secreting adenocarcinomas [5]. Hence, high molecular mucin markers such as CA 125 and CA 19–9 can be important markers, whereas the elevated D-dimer and C-reactive protein levels are reported to be useful for diagnosis of Trousseau’s syndrome in patients with cerebral embolism [6].

In patients with Trousseau’s syndrome, the primary approach for the treatment of thrombosis is total resection of the tumor. Despite being admitted to our hospital 17 days after the cerebral infarction, the semi-radical surgery was scheduled for the 31th day. While performing the surgery at the earliest might be preferable to halt the progression of systemic thrombosis, the risk of cardiovascular complications, including hemorrhagic stroke, is much higher in patients undergoing elective non-cardiac surgery within 3 months after the cerebral infarction [7]. Hence, we carefully determined the best time for surgery after consulting with both the neurology and gynecology departments. Considering both the benefits of an earlier operation and the risk of perioperative cerebrovascular complications, we determined that the best time to perform the operation was 1 month (31 days) after the onset of the cerebral infarction.

The pathomechanism of thrombus formation in Trousseau’s syndrome is still unestablished; however, it is generally accepted that heparin administration is the preferred treatment, since heparin inhibits the binding between cancer-secreting mucin and secretin which leads to platelet aggregation [3, 4]. Unless the tumors are radically eliminated, lifelong heparin administration is required. We adopted the peripheral nerve block with IV infusion of fentanyl for postoperative analgesia instead of neuraxial anesthesia. Thus, it was possible to quickly resume anticoagulant therapy, preventing additional perioperative thrombosis. Moreover, the patient did not complain of surgical wound pain all day after surgery, which was presumably attributable to the effects of the peripheral nerve block and the PCA with fentanyl. From POD 1, oral intake of non-steroidal anti-inflammatory drugs and IV infusion of acetaminophen was used to manage pain, and subsequently, PCA with fentanyl was discontinued on POD 2. Our perioperative pain management appeared to be generally successful.

With regard to anesthetic technique, we used desflurane in order to bring rapid emergence from general anesthesia and to perform neurological assessment after surgery. In fact, she emerged in a few minutes after the discontinuation of anesthesia and showed no further neurological deficits. Prolonged low rSO_2_ values have been demonstrated to correlate with perioperative cerebrovascular events [8]. Although the rSO_2_ value might be limited to the information of frontal lobes, we attempted to stabilize respiratory and circulatory dynamics for maintaining the cerebral perfusion and rSO_2_ levels. Consequently, using these meticulous anesthetic management strategies, our patient had a good perioperative course without adverse cerebral complications or recurrent thromboembolism.

In conclusion, early radical surgery and preventing perioperative cerebrovascular complications are both critical in patients with Trousseau’s syndrome. Although the best timing of surgery is still unestablished, our patient with Trousseau’s syndrome successfully underwent gynecological surgery under general anesthesia 1 month after a massive cerebral infarction.

## Data Availability

Not applicable due to patient privacy concerns.

## Abbreviations

APTT: Activated partial thromboplastin time
CA: Carbohydrate antigen
CT: Computed tomography
IV: Intravenous
IVC: Inferior vena cava
MRI: Magnetic resonance imaging
PCA: Patient-controlled analgesia
POD: Postoperative day
TEE: Transesophageal echocardiography
TTE: Transthoracic echocardiography
